# Reduced CD5 on CD8^+^ T Cells in Tumors but Not Lymphoid Organs Is Associated With Increased Activation and Effector Function

**DOI:** 10.3389/fimmu.2020.584937

**Published:** 2021-01-28

**Authors:** Faizah Alotaibi, Mark Vincent, Wei-Ping Min, James Koropatnick

**Affiliations:** ^1^ Department of Microbiology and Immunology, The University of Western Ontario, London, ON, Canada; ^2^ Cancer Research Laboratory Program, London Regional Cancer Program, Lawson Health Research Institute, London, ON, Canada; ^3^ Department of Oncology, The University of Western Ontario, London, ON, Canada

**Keywords:** tumor-infiltrating lymphocytes (TILs), CD5, CD8^+^ T cells, CD4^+^ T cells, activation, exhaustion

## Abstract

CD5, a member of the scavenger receptor cysteine-rich superfamily, is a marker for T cells and a subset of B cells (B1a). CD5 associates with T-cell and B-cell receptors and increased CD5 is an indication of B cell activation. In tumor-infiltrating lymphocytes (TILs) isolated from lung cancer patients, CD5 levels were negatively correlated with anti-tumor activity and tumor‐mediated activation-induced T cell death, suggesting that CD5 could impair activation of anti-tumor T cells. We determined CD5 levels in T cell subsets in different organs in mice bearing syngeneic 4T1 breast tumor homografts and assessed the relationship between CD5 and increased T cell activation and effector function by flow cytometry. We report that T cell CD5 levels were higher in CD4^+^ T cells than in CD8^+^ T cells in 4T1 tumor-bearing mice, and that high CD5 levels on CD4^+^ T cells were maintained in peripheral organs (spleen and lymph nodes). However, both CD4^+^ and CD8^+^ T cells recruited to tumors had reduced CD5 compared to CD4^+^ and CD8^+^ T cells in peripheral organs. In addition, CD5^high^/CD4^+^ T cells and CD5^high^/CD8^+^ T cells from peripheral organs exhibited higher levels of activation and associated effector function compared to CD5^low^/CD4^+^ T cell and CD5^low^/CD8^+^ T cell from the same organs. Interestingly, CD8^+^ T cells among TILs and downregulated CD5 were activated to a higher level, with concomitantly increased effector function markers, than CD8^+^/CD5^high^ TILs. Thus, differential CD5 levels among T cells in tumors and lymphoid organs can be associated with different levels of T cell activation and effector function, suggesting that CD5 may be a therapeutic target for immunotherapeutic activation in cancer therapy.

## Introduction

CD5 was first discovered in the late 1960s and was known as Lyt-1, Ly-A, or Ly-1 (mouse) and T1 or Leu-1 (human) (1). It was initially used to distinguish subpopulations of T and B cells from other immune cells (2, 3). CD5 is a 67 kDa type-I transmembrane glycoprotein and a member of the highly conserved group B scavenger receptor cysteine-rich (SRCR) protein superfamily (4). This superfamily includes more than 30 soluble or membrane-bound receptors with at least one SRCR extracellular domain in common, and with some family members regulating innate and adaptive responses (5) and a role in pathogen-associated molecular pattern (PAMP) recognition (6).

CD5 expression is associated with the antigen-specific receptor complex in both T and B cells (7–9) and is expressed on both αβ and γδ T cell subsets (10). It is detectable at early stage T cell development and during the double negative (CD4^-^/CD8^-^) stage, with levels increasing during development from double negative to single positive (CD4^+^/CD8^+^ or CD8^+^/CD4^+^) (11). It has been shown to participate in fine-tuning positive and negative selection and enhancing development of high-affinity antigen binding (7, 11). CD5 expression has been linked to the strength of T cell receptor signaling (7). CD5 has also been reported to be expressed on a variety of immune cell subtypes including macrophages (12) and dendritic cells (13). It is highly expressed by CD4^+^ and CD8^+^ T cell receptor (TCR) αβ cells (14) and is increased in regulatory T and B cells (15, 16) and promotes induction of Foxp3 expression (17). Furthermore, chronically stimulated T and B cells have increased CD5 (18, 19).

CD5 has been shown to interact with TCR and impair TCR signaling (7, 20). Previous studies have shown a correlation between CD5 and anti-tumor immunity where CD5 knockout mice inoculated with B16F10 melanoma cells had delayed tumor growth compared to wild type mice (21). Furthermore, TILs with low CD5 expression isolated from lung cancer patients exhibited increase cytotoxic activity compared to CD5^+^ TILs (22). However, the correlation between CD5 level expression and T cell activation and effector function in the tumor microenvironment and in peripheral organs is ill-defined and requires further investigation. We determined the *in vivo* relationship between T cell CD5 level and T cell activation and effector function in primary mouse T cells isolated from different organs and syngeneic tumors. We found different patterns of CD5 expression in T cell isolated from different tissues in mice bearing tumor homografts. The differences in CD5 expression patterns correlated with the level of level of T cell activation and effector function in different organs and tumors.

## Materials and Methods

### Mice and Tumor Model

Female BALB/c mice (Jackson Laboratories, Bar Harbor, ME) between 8 and 12 weeks of age were housed in the Animal Care and Veterinary Services Facility at the Victoria Research Building, Lawson Health Research Institute, according to guidelines of the Canadian Council for Animal Care and under the supervision of the Animal Use Subcommittee of the University of Western Ontario. 4T1 mouse breast mouse tumor cells were purchased from the American Type Culture Collection (ATCC, Manassas, VA) and cultured in Dulbecco modified Eagle medium supplemented with 10% fetal bovine serum (FBS)(Invitrogen). All cells were kept at 37°C in 5% CO_2_.

### *In Vivo* Tumor Challenge

Tumor cells (5X10^4^) were counted by Coulter counter and injected subcutaneously into the right flanks of 2-month-old female BALB/c mice and allowed to grow for 21 days before animals were euthanized by CO_2_ inhalation. Single cell suspensions of lymphocytes were obtained from mice by pressing spleens through a 70 μm Falcon Cell Strainer (VWR, Mississauga, ON) into RPMI 1640 medium (GIBCO). Cells were then centrifuged (300xg, 10 mins, 4°C), and erythrocytes were lysed using Ammonium-Chloride-Potassium (ACK) red cell lysis buffer. The resulting live (trypan blue-negative) splenocytes were counted manually (microscope slide) and cultured for further assessment.

### Tumor-Infiltrating Lymphocyte Preparation

TILs were obtained from freshly resected tumor lesions, which were isolated immediately after mice euthanization. Tumors were cut into 2–3 mm^3^ fragments, and each tumor was placed into individual plate of a 6 well plate and incubated in 2 ml of an enzyme digest mix consisting of RPMI1640 complete media containing % fetal bovine serum (FBS)(Invitrogen) and 10 mg ml^−1^ collagenase A (all from Sigma-Aldrich, Gillingham, UK) and incubated for 2 h at room temperature under continuous rotation.

### Splenocyte and Lymphocyte Preparation

Single cell suspensions of lymphocytes from spleens and lymph nodes were obtained from mice by pressing spleens or lymph nodes through a 70 μm Falcon Cell Strainer (VWR, Mississauga, ON) into RPMI 1640 medium (GIBCO). Cells were then centrifuged (300 x g, 10 min, 4°C), and erythrocytes were lysed using Ammonium-Chloride-Potassium (ACK) red cell lysis buffer. The resulting live (trypan blue-negative) splenocytes and lymphocytes were counted manually (microscope slide) and stained directly or cultured for further assessment.

### Intracellular IFN-γ Staining

To measure IFNγ in CD8^+^ T cells, ICS was restricted to detection of IFNγ (a cytokine produced by CD8^+^ cells upon activation (23). Cells from spleens, lymph nodes, or TILs isolated from 4T1 tumor-bearing BALB/c mice were isolated and single cell suspensions prepared as described. Cells were then seeded into U-bottom 96-well plates precoated with 5 μg/ml anti-CD3 antibody, in RPMI media containing 10% FBS and soluble anti-CD28 antibody (Clone: 37.51. BD Biosciences, 2 μg/ml). Cells were incubated at 37°C overnight in 5% CO_2_. Brefeldin A (10 μg/ml) was added to retain secretion of IFNγ in the Golgi apparatus. After 3 h, cells were stained with extracellular markers including anti-mouse PerCP-Cy5.5 CD8a (clone 53-6.7) MAb and anti-mouse PE-Cy7 CD5 (clone 53-6.7) MAb, (Biolegend, San Diego, CA)(1 μg/ml in 50 μl FACS buffer) on ice and incubated in the dark for 30 min. The samples were washed twice and fixed in 2% paraformaldehyde (50 μl). To detect CD8^+^ T cell activation, samples were stained for 60 min with anti-mouse PE-IFN-γ clone (XMG1.2) (1 μg/ml in intracellular staining permeabilization wash buffer) (Biolegend, San Diego, CA). Samples were then washed and harvested in FACS buffer for flow cytometry. Flow cytometric data were analyzed using Flowjo software (BD Bioscience).

## Flow Cytometry

To assess the levels of CD69, CD5, PD-1, CD107a, CD137, and CTLA-4 in CD4^+^ and CD8^+^ T cells, the following antibodies were used for flow cytometry: anti-mouse Brilliant Violet 711™ CD3 (clone 17A2), anti-mouse Alexa Fluor^®^ 700 CD4 (clone GK1.5) anti-mouse FITC CD8 (clone 53-5.8), anti-mouse PerCP-Cy5.5 CD8a (clone 53-6.7) anti-mouse FITC CD5 (clone 53-7.3), anti-mouse PE-Cy7 CD5 (clone 53-6.7) anti-mouse PE/Dazzle™ 594 CD279 (PD-1) clone (29F.1A12), anti-mouse PE CD69 (clone H1.2F3), anti-mouse BV421 CD107a clone (1D4B), anti-mouse FITC CD137, and anti-mouse BV605 CTLA-4 clone (UC10-4B9)(Biolegend, San Diego, CA). Flow cytometry was performed using a flow cytometer LSR II (BD Bioscience) and data analyzed using Flowjo software (BD Bioscience). To assess the level of the indicated markers, spleens, lymph nodes and tumor were collected from either tumor-naïve mice or tumor-bearing mice 21 days after tumor cell injection. Single cell suspension was prepared as previously described. Cells were then stained with Zombie NIR™ Fixable Viability Kit (Biolegend, San Diego, CA) to exclude dead cells. Then cells were incubated with purified anti-mouse CD16/32 antibody (Clone 93)(Biolegend, San Diego, CA) for 15 min at 21°C in the dark to block CD16/CD32 interactions with the Fc domain of immunoglobulins, following with appropriate antibodies for 25 mins on ice in the dark, washed twice with FACS staining buffer, suspended in 0.5 ml FACS staining buffer, and analyzed by flow cytometry.

### Statistical Analysis

Statistical differences were assessed using a Student’s paired one-tailed *t*-test (GraphPad Prism 8.2.1). Data points indicate means of *n* values ± standard deviation (SD). Differences between data sets where *p* ≤ 0.05 were considered to be significant. Asterisks represent statistical significance.

## Results

### Differential CD5 Expression Among Organs and T Cell Subsets

**Figure 1:**
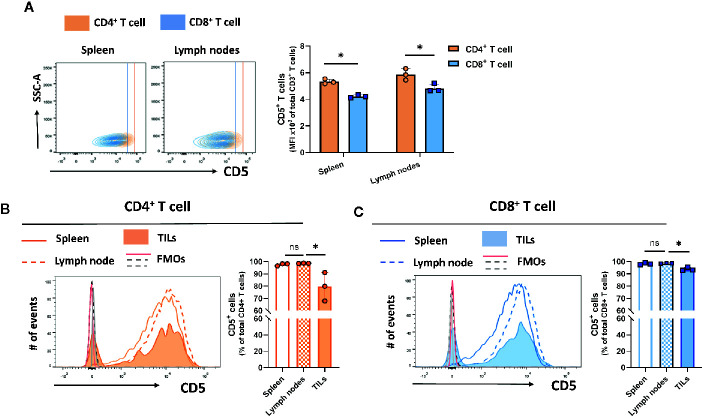
CD5 levels in T cell subsets and lymphoid organs. T cells isolated from 4T1-harboring BALB/c mice were stained with fluorescence-conjugated anti-CD3, anti-CD4, anti-CD8, and anti-CD5 MAbs. **(A)** CD5 levels on CD8^+^ T cells and CD4^+^ T cells in lymph nodes and spleen. **(B)** The fraction of CD5^+^/CD4^+^ T cell solated from the lymph nodes, spleen, and TILs. **(C)** The fraction of CD5^+^/CD8^+^ T cells isolated from the lymph nodes, spleen, and TILs. Data are shown as means ± SD of 3 mice per group and from one representative experiment of three independent experiments. FMO, Fluorescence Minus One Control; TILs, tumor-infiltrating lymphocytes; MFI, mean fluorescence intensity; ns, not significant. *p < 0.05 (Student’s paired two-tailed t-test).

CD5 is expressed on the majority of T cells (24). However, differences in the levels of expression among T cell subsets isolated from different peripheral organs are not well-described. To determine the level of CD5 among T cell subsets and within peripheral organs and tumor cells, mice subcutaneously implanted with syngeneic triple-negative 4T1 breast tumors were euthanized on day 21 after implantation and spleen, lymph nodes, and tumor tissue were harvested and processed to generate single cell suspensions containing immune cells. The recovered cells were stained for CD5 and surface markers of T cell subsets. The result shows CD4^+^ T cells in spleen and lymph nodes had higher levels of surface CD5 than CD8^+^ T cells in those tissues (**Figure 1A**). There was no significant difference between CD5 levels in CD4^+^ T cells from spleen and lymph nodes (**Figure 1B**), and no significant difference in CD5 levels between CD8^+^ T cells from spleen and lymph nodes (**Figure 1C**). Interestingly, a small fraction of both CD4^+^ and CD8^+^ T cells infiltrated into tumors (TILs) had lower CD5 surface levels than CD4^+^ and CD8^+^ T cells from lymph nodes and spleen (**Figures 1B, C**). Taken together, these data indicate that CD5 levels are significantly higher in CD4^+^ T cells compared to CD8^+^ T cells. Furthermore, a fraction of both CD4^+^ T cell and CD8^+^ T cells recruited to the tumor microenvironment have lower CD5 levels than T cells in lymph and spleen, either because CD5 is downregulated by the tumor microenvironment or T cells with lower CD5 are preferentially infiltrated into tumors. We also determined whether CD5 levels on T cells were affected by the presence of tumor homografts. T cells were isolated from lymph nodes of naïve mice and 4T1-harboring mice on day 21 after tumor cell implantation. Cells were stained with anti-CD5 MAb in addition to antibodies targeting CD3, CD4, and CD8. The levels of CD5 on the surfaces of naïve T cell and T cell from 4T1 tumor homograft-harboring mice showed different patterns of expression. CD5 levels on both CD4^+^ T cells (**Supplemental Figure 2A**) and CD8^+^ T cells (**Supplemental Figure 2B**) were significantly higher in lymph nodes of mice harboring 4T1 tumors compared to naïve mice. Furthermore, as previously reported by us (25), CD5 levels on CD8^+^ T cell splenocytes were significantly increased after TCR/CD3 stimulation using *ex vivo* treatment with anti-CD3/anti-CD28 MAbs compared to non-stimulated CD8^+^ T splenocytes (25). Together, these results reveal that the presence of poorly immunogenic tumor homografts in mice leads to elevated CD5 on T cells in lymph nodes, similar to the increase in CD5 seen after *ex vivo* stimulation of the TCR/CD3 complex on CD8^+^ T cells.

### CD5^high^ T Cells in Spleen and Lymph Nodes Exhibit Increased Activation

**Figure 2:**
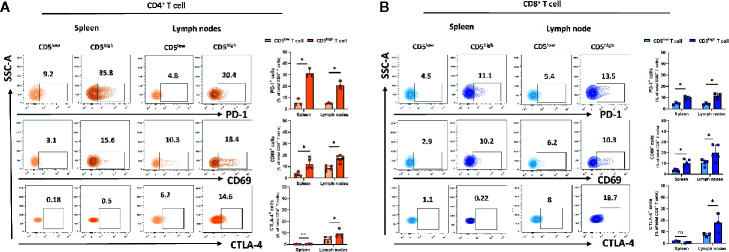
CD5 levels correlate with PD-1, CD69, and CTLA-4 in lymphoid organs. Splenocytes and lymphocytes isolated from 4T1-harboring BALB/c mice were stained with fluorescence-conjugated antibodies to assess PD-1, CD69, and CTLA-4 in correlation to CD5. **(A)** The fraction of PD-1^+^/CD5^high^/CD4^+^, CD69^+^/CD5^high^/CD4^+^, CTLA-4^+^/CD5^high^/CD4^+^ T, PD-1^+^/CD5^low^/CD4^+^, CD69^+^/CD5^low^/CD4^+^, and CTLA-4^+^/CD5^low^/CD4^+^ T cells in spleen and lymph nodes, respectively. **(B)** The fraction of PD-1^+^/CD5^high^/CD8^+^, CD69^+^/CD5^high^/CD8^+^, CTLA-4^+^/CD5^high^/CD8^+^ *vs* PD-1^+^/CD5^low^/CD8^+^, CD69^+^/CD5^low^/CD8^+^, and CTLA-4^+^/CD5^low^/CD8^+^ T cells in spleen and lymph nodes, respectively. Data are shown as means ± SD of 5 or 3 mice per group and from one representative experiment of three independent experiments. *p < 0.05 (Student’s paired two-tailed t-test. ns, not significant).

The increased level of CD5 on T cells upon TCR/CD3 stimulation by monoclonal antibody treatment (25) or by exposure to poorly immunogenic 4T1 tumor antigen reported here suggests that CD5 level may be directly increased by that activation. To address how CD5 levels may be associated with T cell activation, a gating strategy was applied to determine the activation level of CD5^high^ T cells and CD5^low^ T cells based on the level of the T cell activation markers that are induced upon T cell activation, including CD69 (26), PD-1 (27), and CTLA-4 (28) (See Gating Strategy, **Supplemental Figure 1**). Mice were challenged by subcutaneous injection of 4T1 tumor cells 21 days prior to euthanasia and collection of lymph nodes and spleens. T cells isolated from those organs were stained with anti-CD69, anti-PD-1, anti-CTLA-4 and anti-CD5 MAbs in addition to antibodies against T cell markers (anti-CD3/anti-CD8/anti-CD4 MAbs). The results show that the fraction of CD69^+^/CD5^high^/CD4^+^ T cells and PD-1^+^/CD5^high^/CD4^+^ T cells in spleen and lymph nodes, and CTLA-4^+^/CD5^high^/CD4^+^ T cells in lymph nodes, were significantly higher compared to the fraction of CD69^+^/CD5^low^/CD4^+^ T cells, PD-1^+^/CD5^low^/CD4^+^ T cells, and CTLA-4^+^/CD5^low^/CD4^+^ T cells, respectively (**Figure 2A**). Similarly, the fraction of CD69^+^/CD5^high^/CD8^+^ T cells and PD-1^+^/CD5^high^/CD8^+^ T cells in spleen and lymph nodes, and CTLA-4^+^/CD5^high^/CD4^+^ T cells in lymph nodes, were significantly higher than the fraction of CD69^+^/CD5^low^/CD8^+^ T cells, PD-1^+^/CD5^low^/CD8^+^ T cells, and CTLA-4^+^/CD5^low^/CD8^+^ T cells, respectively (**Figure 2B**). Collectively, these data suggest a correlation between CD5 level and T cell activation in peripheral organs.

### CD5^-/low^ T Cells Display Increased Activation in the Tumor Microenvironment

**Figure 3:**
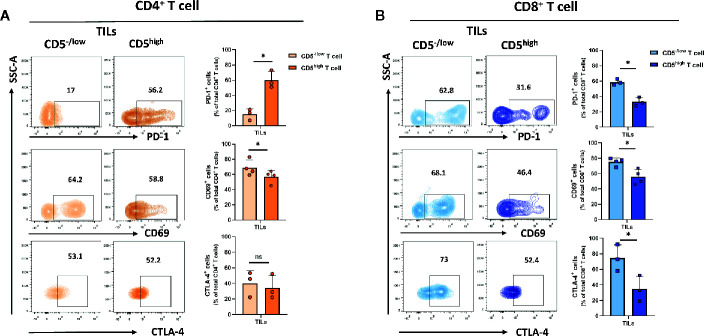
CD5 levels and their correlation with PD-1, CD69 and CTLA-4 in TILs. TILs isolated from 4T1-harboring BALB/c mice were stained with fluorescence-conjugated antibodies to assess PD-1, CD69 and CTLA-4 in correlation with CD5. **(A)** The fraction of PD-1^+^/CD5^high^/CD4^+^, CD69^+^/CD5^high^/CD4^+^, CTLA-4^+^/CD5^high^/CD4^+^ *vs* PD-1^+^/CD5^low^/CD4^+^, CD69^+^/CD5^low^/CD4^+^, and CTLA-4^+^/CD5^low^/CD4^+^ TILs. **(B)** The fraction of PD-1^+^/CD5^high^/CD8^+^, CD69^+^/CD5^high^/CD8^+^, CTLA-4^+^/CD5^high^/CD8^+^ *vs*PD-1^+^/CD5^low^/CD8^+^, CD69^+^/CD5^low^/CD8^+^, and CTLA-4^+^/CD5^low^/CD8^+^ TILs. Data are shown as means ± SD of 4 or 3 mice per group and from one representative experiment of three independent experiments. *p < 0.05 (Student’s paired two-tailed t-test. ns, not significant).

A small fraction of T cells recruited to the tumor microenvironment display reduced CD5 levels compared to T cells in lymph nodes and spleens. To determine the level of activation of CD5^high^ and CD5^-/low^ tumor-infiltrating T cells, the level of the activation markers including CD69 (26), PD-1 (27), and CTLA-4 (28) in both CD4^+^ and CD8^+^ T cell subpopulations in T cells isolated from 4T1 tumors excised from mice at 21 days following subcutaneous implantation of tumor cells was determined. The data show that the fraction of CD69^+^/CD5^−/low^/CD4^+^ TILs was significantly lower than CD69^+^/CD5^high^/CD4^+^ TILs, suggesting a lower level of activation associated with low CD5 in CD4^+^ TILs (**Figure 3A**). On the other hand, the fraction of PD-1^+^/CD5^−/low^/CD4^+^ TILs was significantly higher than the fraction of PD-1^+^/CD5^high^/CD4^+^ TILs, suggesting that at least some aspects of activation associated with PD-1 expression are increased in CD5^-/low^ TILs (**Figure 3A**) (**Supplementary Figure 3** for CD69 and CD5 co-stained in CD8^+^ T cell from lymph nodes and TILs). No differences between the fraction of CTLA-4^+^/CD5^−/low^/CD4^+^ TILs and CTLA-4^+^/CD5^high^/CD4^+^ TILs were observed (**Figure 3A**). On the other hand, all three measures of activation in CD8^+^ TILs were strongly associated with low CD5: the fraction of CD69^+^/CD5^−/low^/CD8^+^ TILs, PD-1^+^/CD5^−/low^/CD8^+^ TILs, and CTLA-4^+^/CD5^-/low^/CD8^+^ TILs were significantly higher than the fraction of CD69^+^/CD5^high^/CD8^+^ TILs, PD-1^+^/CD5^high^/CD8^+^ TILs, and CTLA-4^+^/CD5^high^/CD8^+^ TILs, respectively (**Figure 3B**). This suggests that the fraction of CD8^+^ TILs with reduced CD5 levels in the tumor microenvironment have higher levels of activation than CD5^high^/CD8^+^ TILs, while the situation with CD4^+^ TILs is more complex. To further explore activation in both CD4^+^ and CD8^+^ TILs, we assessed CD69^+^ and CD69^-^ subpopulations within CD5^-/low^/CD4^+^ and CD5^-/low^/CD8^+^ TILs for their levels of CD3 (key receptor in TCR signaling) and CD107a (surrogate marker for CTL degranulation) (29). We found higher levels of both CD3 and CD107a within the CD69^+^ population than within CD69^-^ TILs, in both CD5^-/low^/CD4^+^ and CD5^-/low^/CD8^+^ TILs (**Supplementary Figure 4**). These data further support an association between low CD5 and uniformly increased activation in CD8^+^ TILs, and some aspects of activation in CD4^+^ TILs.

### CD5^high^ T Cells in Spleen and Lymph Nodes Exhibit Increased Effector Function

**Figure 4:**
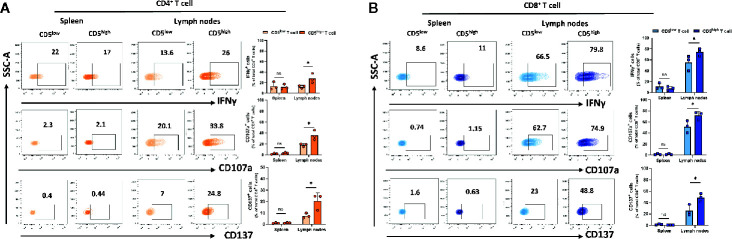
CD5 levels and their correlation with IFNγ, CD107a and CD137 in lymphoid organs. Splenocytes and lymphocytes isolated from 4T1-harboring BALB/c mice were stained with fluorescence-conjugated antibodies to assess IFNγ, CD107a and CD137 in correlation with CD5. **(A)** The fraction of IFNγ^+^/CD5^high^/CD4^+^, CD107a^+^/CD5^high^/CD4^+^, CD137^+^/CD5^high^/CD4^+^ *vs* IFNγ^+^/CD5^low^/CD4^+^, CD107a^+^/CD5^low^/CD4^+^, and CD137^+^/CD5^low^/CD4^+^ T cell in spleen and lymph nodes, respectively. **(B)** The fraction of IFNγ^+^/CD5^high^/CD8^+^, CD107a^+^/CD5^high^/CD8^+^, CD137^+^/CD5^high^/CD8^+^ *vs* IFNγ ^+^/CD5^low^/CD8^+^, CD107a ^+^/CD5^low^/CD8^+^, and CD137^+^/CD5^low^/CD8^+^ T cells in spleen and lymph nodes, respectively. Data are shown as means ± SD of 3 mice per group and from one representative experiment from two independent experiments. *p < 0.05 (Student’s unpaired two-tailed t-test. ns, not significant).

Upon activation, T cells express high levels of CD5 and this increase was associated with increased activation markers. However, these activation markers can also be expressed by exhausted T cells. To determine whether CD5^high^ T cells in spleen and lymph nodes exhibit increased exhaustion or increased effector function compared to CD5^low^ T cells, T cells from spleen and lymph nodes of 4T1 tumor-harboring mice were isolated on day 21 following subcutaneous implantation of the tumor cells. We measured the secretion of IFNγ and a surrogate marker for CTL degranulation: the level of CD107a (29). In addition, antigen-specific activation of T cells was assessed using a surrogate marker for antigen-specific T cell activation: CD137, a member of the TNFR family with costimulatory function (30). The results show that the fraction of IFNγ ^+^/CD5^high^/CD4^+^ T cells, CD107a^+^/CD5^high^/CD4^+^ T cells, and CD137^+^/CD5^high^/CD4^+^ T cells were substantially higher than the fraction of IFNγ ^+^/CD5^low^/CD4^+^ T cells, CD107a^+^/CD5^low^/CD4^+^ T cells, and CD137^+^/CD5^low^/CD4^+^ T cells in lymph nodes (**Figure 4A**). Similarly, the fraction of IFNγ ^+^/CD5^high^/CD8^+^ T cells, CD107a^+^/CD5^high^/CD8^+^ T cells, and CD137^+^/CD5^high^/CD8^+^ T cells were significantly higher than the fraction of IFNγ ^+^/CD5^low^/CD4^+^ T cells, CD107a^+^/CD5^low^/CD4^+^ T cells and CD137^+^/CD5^low^/CD4^+^ T cells in lymph nodes (**Figure 4B**). The same T cell subsets in spleens did not show similar increases in markers of effector function (**Figures 4A, B**). Collectively, these data suggest that elevated CD5 on T cells in lymph nodes, but not spleens, is associated with T cell effector function. The question of whether, among activated T cells, those that were CD5^-/low^ were more effectively activated is relevant and reported below.

### CD5^-/low^/CD8^+^ T Cells Display Increased Effector Function in the Tumor Microenvironment

**Figure 5:**
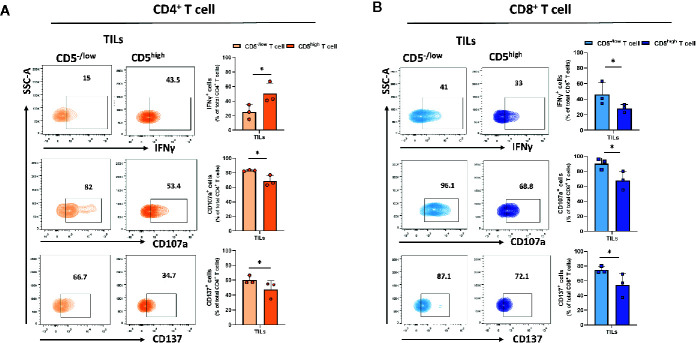
CD5^-/low^/CD4^+^ and CD5^-/low^/CD8^+^ TILs have increased effector function (IFNγ, CD107a, and CD137 expression) compared to CD5^high^ TILs. TILs isolated from 4T1-harboring BALB/c mice were stained with fluorescence-conjugated antibodies to assess IFNγ, CD107a and CD137 in relationship with CD5. **(A)** The fraction of IFNγ^+^/CD5^high^/CD4^+^, CD107a^+^/CD5^high^/CD4^+^, CD137^+^/CD5^high^/CD4^+^ *vs* IFNγ^+^/CD5^-/low^/CD4^+^, CD107a^+^/CD5 ^-/low/^CD4^+^, and CD137^+^/CD5 ^-/low/^CD4^+^ TILs, respectively. **(B)** The fraction of IFNγ^+^/CD5^high^/CD8^+^, CD107a^+^/CD5^high^/CD8^+^, CD137^+^/CD5^high^/CD8^+^ *vs* IFNγ ^+^/CD5^-/low^/CD8^+^, CD107a ^+^/CD5^-/low^/CD8^+^, and CD137^+^/CD5^-/low/^CD8^+^ TILs, respectively. Data are shown as means ± SD of 4 or 3 mice per group and from one representative experiment of two independent experiments. *p < 0.05 (Student’s paired two-tailed t-test).

**Figure 6:**
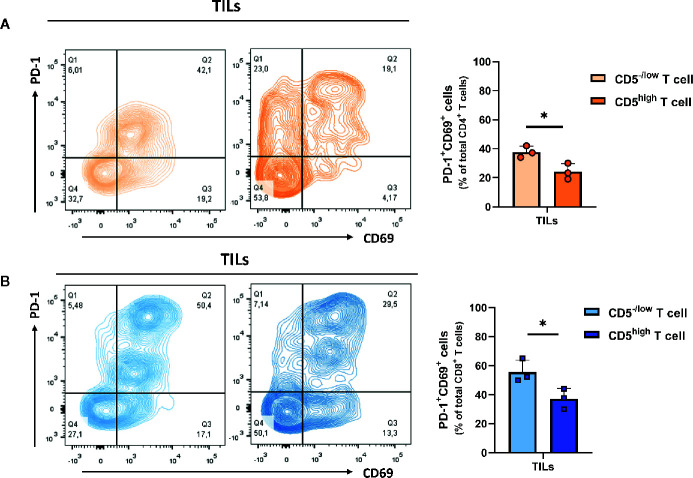
Both CD5^-/low^/CD4^+^ and CD5^-/low^/CD8^+^ TILs have increased effector function (co-expression of PD-1 and CD69). **(A)** The fraction of PD-1^+^/CD69^+^/CD5^-/low^/CD4^+^ TILs and PD-1^+^/CD69^+^/CD5^high^/CD4^+^ TILs. **(B)** The fraction of PD-1^+^/CD69^+^/CD5^-/low^/CD8^+^ TILs and PD-1^+^/CD69^+^/CD5^high^/CD8^+^ TILs. Data are shown as means ± SD of 3 mice per group and from one representative experiment of three independent experiments. *p < 0.05 (Student’s paired two-tailed t-test).

Downregulation of CD5 was associated with an increased fraction of activated CD4^+^ and CD8^+^ T cells in the tumor microenvironment. To determine the effector function in CD5^-/low^CD4^+^ *vs* CD5^high^CD4^+^ TILs and in CD5^-/low^/CD8^+^ *vs* CD5^high^/CD8^+^ TILs, TILs were stained with anti-IFNγ, anti-CD107a, and anti-CD137 MAbs and with antibodies to detect CD5, CD4, CD8, and CD3. The results show a significantly elevated fraction of CD107a^+^/CD5^-/low^/CD4^+^ TILs and CD137^+^/CD5^-/low^/CD4^+^ TILs compared to CD107a^+^/CD5^high^/CD4^+^ TILs and of CD137^+^/CD5^high^/CD4^+^ TILs, respectively (**Figure 5A**); and the fraction of IFNγ ^+^/CD5^high^/CD4^+^ TILs was significantly higher compared to IFNγ^+^/CD5^-/low^/CD4^+^ TILs (**Figure 5A**). Furthermore, CD8^+^ TILs had an increased fraction of IFNγ^+^/CD5^-/low^/CD8^+^ TILs, CD107a^+^/CD5^-/low^/CD8^+^ TILs, and CD137^+^/CD5^-/low^/CD8^+^ TILs compared to IFNγ^+^/CD5^high^/CD8^+^ TILs, CD107a^+^/CD5^high^/CD8^+^ TILs, and CD137^+^/CD5^high^/CD8^+^ TILs, respectively (**Figure 5B**). These data suggest that, among both CD4^+^ and CD8^+^ TILs, the CD5^-/low^ fraction have increased effector function compared to the CD5^high^ fraction. We further looked at co-expression of CD69 and PD-1 as another measurement for effector T cell function (31). The results show an increased fraction of CD5^-/low^/CD69^+^/PD-1^+^/CD8^+^ TILs and CD5^-/low^/CD69^+^/PD-1^+^/CD4^+^ TILs compared to CD5^high^/CD69^+^/PD-1^+^/CD8^+^ TILs and CD5^high^/CD69^+^/PD-1^+^/CD4^+^ TILs, respectively (**Figures 6A, B**).

## Discussion

Our understanding of CD5 function has progressed over the past few decades. However, we still lack clear understanding of the correlation between CD5 expression and T cell function. We investigated the pattern of CD5 expression in T cells and their subsets in spleen, lymph nodes, and syngeneic tumors in mice, and the relationship between the pattern of expression and T cell activation and effector function. First, we determined CD5 levels in T cell subsets isolated from lymphoid organs and the tumor microenvironment. CD5 expression was observed in T cell subsets including CD4^+^ T cells and CD8^+^ T cells: CD4^+^ T cells were found to express greater amounts of CD5 than CD8^+^ T cells. CD5 has been reported to be highly expressed in regulatory T cells (32) and, because a subset of CD4^+^ T cells are T_regs_, it is not surprising to observe higher CD5 levels in CD4^+^ T cells compared to CD8^+^ T cells. CD5 is associated with the TCR/CD3 complex and BCR and has been shown to negatively regulate the TCR signaling pathway (7). Elevated CD5 is associated with B cell activation (33) and is found to be expressed on certain lymphoid tumors (9). Our data reveal that T cell CD5 levels are enhanced following recognition of specific target [exposure to tumor antigen as reported here or stimulation with anti-CD3 MAb) (25)]. Our results indicate a clear correlation between CD5 and T cell activation and effector function. Considering that CD5 is associated with TCR and BCR (24, 34), this agrees with a published report that CD5 is indicator of B cell activation (33). Furthermore, we report that T cell subsets from mouse spleen and lymph nodes display similar CD5 levels. However, a small fraction of both CD4^+^ and CD8^+^ T cell subsets recruited to the tumor microenvironment had a reduced level of CD5. Although we did not distinguish between the possibilities that T cells with reduced CD5 prior to infiltration into tumors were preferentially recruited into tumors, or that T cells with elevated CD5 infiltrated tumors but had their CD5 levels reduced within the tumor microenvironment, CD5 levels in T cells have been reported to be induced in correlation with the intensity of antigen recognition by TCR (24). Mouse 4T1 tumors are poorly immunogenic and highly metastatic (35), providing a context conducive to poor antigen recognition leading to low CD5 on T cells within those tumors. Furthermore, a previous report indicated that CD5 is reduced by cytokine priming by molecules including IL-7 and IL-21 (36). IL-7 is produced by non-hematopoietic cells including stromal cells, intestinal epithelial cells, and keratinocytes and often in response to inflammation and stimulation by cytokines including IFNγ and tumor necrosis factor-a (TNF-a) (37, 38) while IL-21 is mainly produced by T cells (39). It is possible that the presence of IL-7 and IL-21 in the tumor microenvironment reduced CD5 by modulating transcriptional and/or posttranscriptional control mechanisms (36) although that possibility is not specifically addressed by our data. That, along with our observation of reduced CD5 on T cells within the tumor microenvironment and our previous report that experimental reduction of CD5 leads to increased CD8^+^ T cell activation (25), collectively suggest that T cells with low CD5 within tumors (due wholly or in part to the poorly immunogenic 4T1 tumor microenvironment) may have increased sensitivity to poorly immunogenic 4T1 tumor antigens. These results demonstrated that CD5 may be downregulated in T cells based on the TCR/CD3 signaling intensity and cytokines present in the tumor microenvironment.

We further tested T cell activation in relation to CD5 levels in spleens, lymph nodes, and 4T1 tumors. In lymphoid organs CD69^+^/CD5^high^/CD4^+^, PD-1^+^/CD5^high^/CD4^+^, and CTLA-4^+^/CD5^high^/CD4^+^ T cells, and CD69^+^/CD5^high^/CD8^+^, PD-1^+^/CD5^high^/CD8^+^, and CTLA-4^+^/CD5^high^/CD8^+^ T cells, were significantly higher than their CD5^-/low^/CD4^+^ and CD5^-/low^/CD8^+^ T cell counterparts in spleens and lymph nodes, respectively. This suggests that CD5 levels increase on T cells as TCR stimulation proceeds and T cells undergo activation (as evidenced by increased CD69, PD-1, and CTLA-4). On the other hand, among CD4^+^ and CD8^+^ T cells in the tumor microenvironment (TILs), those with low/undetectable CD5 exhibited higher levels of measures of activation compared to CD5^high^ TILs although CD8^+^ TILs displayed a more uniformly high display of markers of activation/effector activity (elevated PD-1, CD69, CTLA-4, IFN*γ*, CD107a, and CD137) associated with low CD5 than did CD4^+^ TILs (elevated CD69, CD107a, and CD137 but lower PD-1 and no significant change in CTLA-4): an observation suggesting a more complex relationship between activation and CD5 in CD4^+^ TILs. Regardless, an increased level of activation in T cells is in accord with our previous report that inhibition of CD5 on T cells leads to enhanced T cell activation and proliferation (25) and suggesting that T cells with low CD5 are selected for increased presence within 4T1 tumors either by preferential recruitment of CD5^-/low^ T cells from peripheral tissues into tumors or preferential activation of CD5^-/low^ T cells once they migrate into tumors. Furthermore, CD5 has been reported to protect tumor-reactive circulating T cells from activation-induced cell death (AICD) following recognition of autologous tumor (40). Reduced CD5 levels on T cells within the 4T1 tumor microenvironment may be evidence of selection of T cells with naturally-occurring low CD5 for enhanced activation and proliferation within tumors: such selection would promote interaction between T cells and tumor cells, enhance the TCR signaling response, and result in increased activation as shown by increased CD69, PD-1 and CTLA-4 expression. This is consistent with a report of increased anti-tumor cytolytic activity in T cells with low CD5 levels where TILs isolated from lung cancer patients exhibited differential anti-tumor activity where CD5 levels were negatively correlated with that anti-tumor activity (22). The mechanism regulating expression *in vivo* is not well understood and it is difficult, with our present level of understanding, to determine the reasons underlying our observation of CD8^+^ T cells with reduced CD5 in the tumor microenvironment compared to T cells from lymph node and spleen. Regardless of that, low CD5 on T cells within tumors can conceivably lead to increased anti-tumor immune activity. There is a more complex situation with CD4^+^ TILs where we did not observe low CD5 in an increased fraction of the CTLA-4^+^ and PD-1^+^ subsets similar to CD8^+^ TILs with low CD5. This may be due to the fact that CTLA-4 and PD-1 are considered to be both activation and exhaustion markers and are upregulated when T cells are chronically exposed to non-self-tumor antigens (as is the case in our model of 4T1 breast tumor growth). This role in host defense is fulfilled by CD8^+^ T cells, making them preferentially susceptible to CTLA-4 and PD-1 upregulation after exposure to tumor antigen. CD4^+^ T cells, on the other hand, do not recognize tumor cells directly due to the lack of MHC II on solid tumor cells (41). Therefore, they may not upregulate exhaustion/activation markers similar to CD8^+^ TILs when they are activated, due to limited interaction with tumor cells. Although this explanation is consistent with our observations it requires further investigation and supporting evidence.

Expression of CD69, PD-1, and CTLA-4 by T cells are measures of T cell activation but can also be measures of T cell exhaustion. To further investigate T cell activation in relation to CD5, we measured the levels of IFNγ, CD107a, and CD137, all of which are highly expressed by effector T cells. We found that, in lymph nodes, CD5^high^ T cells exhibited a high fraction of IFNγ^+^, CD107a^+^ and CD137^+^ T cells compared to CD5^low^ T cells in both CD4^+^ and CD8^+^ subsets. Furthermore, we observed a minimal level of effector function in T cells from the spleens: perhaps unsurprising in view of the presence of myeloid-derived suppressor cells {MDSCs, known to suppress T cell function [reviewed in (42).]} in spleens of 4T1-harboring mice (43). To investigate further whether CD5^-/low^ TILs display increased levels of exhaustion/effector markers we measured IFNγ, CD107a, and CD137 in association with CD5 levels in TILs. We observed that CD5 was inversely associated with T cell effector function: CD5^-/low^/CD4^+^ TILs encompassed a high fraction of CD107a^+^ and CD137^+^ TILs and, similarly, CD5^-/low^/CD8^+^ TILs encompassed a higher fraction of CD107a^+^, CD137^+^, and IFNγ^+^ TILs compared to CD5^high^/CD8^+^ TILs. This suggests the reduced CD5 in TILs, particularly in CD8^+^ TILs, is associated with increased effector function and enhanced anti-tumor immunity. It has been reported previously that CD5 on T cells is inversely correlated with the affinity and avidity of TCR binding to epitopes presented by MHC molecules (24, 44). T cells in lymphoid organs are primed to activation by tumor antigen by professional antigen-presenting cells which present tumor epitopes after binding to MHC molecules. Because CD5 negatively regulates TCR signaling, activation leads to increased CD5 to limit activation and prevent activation-induce cell death (AICD). However, in the tumor microenvironment, antigen presentation by tumor cells is less potent than presentation by professional antigen-presenting cells (45): under those circumstances, reduced CD5 levels could enhance TCR recognition to tumor epitopes and enhance T cell activation and anti-tumor activity. We propose, therefore, that downregulation of CD5 in TILs is likely to enhance the activation and effector function of TILs in close proximity to tumors.

In summary, our data suggest that CD5 levels on T cells (particularly CD8^+^ T cells) are correlated with T cell activation and effector function, as evidenced by increase expression of activation and effector markers: that correlation supports a role for CD5 in T cell survival. It is known that T cell subsets express different levels of CD5 (32). However, we report here for the first time that CD5 levels in these subsets is different in lymphoid organs and the tumor microenvironment and that CD5^-/low^ TILs exhibit increased markers of activation and effector function, unlike CD5^low^ T cells in lymphoid organs. CD5 is induced based on the intensity of the TCR-MHC-1 interaction and poorly immunogenic tumor microenvironments that can lead to T cells with reduced CD5: a situation promoting increased TCR-MHC-I interaction and enhanced T cell activation. A causal role for CD5 in T cell activation, supported by evidence presented here and by us previously (25), forms the basis to propose targeting of CD5 as a therapeutic intervention to enhance anti-tumor immunity. Immune checkpoint blockade is a current immunotherapeutic approach in cancer that is most effective in patients with an increased fraction of PD-1^+^ cells (46). Therapeutic interventions that lead to enhanced activation of T cells in the tumor microenvironment (for example, interventions to reduce CD5) may be suitable components of combination therapy that include anti-CD5 treatment with immune checkpoint blockade: anti-CD5 would lead to increased PD-1 on tumor-resident T cells that, in turn, would lead to a situation promoting the effectiveness of anti-PD-1 immune checkpoint blockade. The current study provides evidence for a critical role for CD5 in regulating PD-1 expression in tumor-associated CD8^+^ T cells and suggests a therapeutic approach to sensitize T cell to immune checkpoint blockade.

## Data Availability Statement

The original contributions presented in the study are included in the article/**Supplementary Material**; further inquiries can be directed to the corresponding author.

## Ethics Statement

The animal study was reviewed and approved by the Animal Use Subcommittee of the University of Western Ontario.

## Author Contributions

FA and JK designed the research strategy. FA performed the research. FA, MV, WM, and JK proofread the paper. FA and JK analyzed the data. and FA and JK wrote the paper. All authors contributed to the article and approved the submitted version.

## Funding

This research has been funded by grants to JK from the Canadian Institutes of Health Research (CIHR) and the London Regional Cancer Program, and a King Abdullah Scholarship to FA.

## Conflict of Interest

The authors declare that the research was conducted in the absence of any commercial or financial relationships that could be construed as a potential conflict of interest.

## Supplementary Material

Supplementary Figure 1 Gating strategy for CD5^high^ and CD5^-/low^ T cell. Cells were gated based on their forward scatter and side scatter. Duplicate and dead cells were excluded then CD3^+^ cell were gated followed by CD4^-^ and CD8^-^ cell gating. CD5 on each CD4^+^ T cells and CD8^+^ T cells was gated 30% from the right as high expression and 30% from the left as low expression. Further analyses were carried out to look at CD69 and PD-1 surface markers.

Supplementary Figure 2 CD5 expression increased upon activation. Lymphocytes isolated from naïve mice or 4T1 tumor-harboring BALB/c mice were stained with MAbs targeting CD4, CD8, and CD5. **(A)** The level of CD5 on CD4^+^ T cells isolated from lymph nodes of naïve mice and 4T1 tumor-harboring mice. **(B)** The level of CD5 on CD8^+^ T cells isolated from lymph nodes of naïve mice and 4T1 tumor-harboring mice. Data are shown as means ± SD of 3 mice per group and from one representative experiment from three independent experiments. FMO: Fluorescence Minus One Control. MFI: mean fluorescence intensity. NS, not significant. *p < 0.05 (Student’s paired two-tailed t-test).

Supplementary Figure 3 Co-stain plot for CD69 and CD5 in CD8^+^ T cells isolated from lymph nodes and tumor-infiltrating lymphocytes (TILs).

Supplementary Figure 4 CD107a and CD3 in CD69^+^ and a CD69^-^ populations within CD5^-/low^/CD4^+^ TILs **(A)** and CD5^-/low^/CD8^+^ TILs **(B)**.
