# Supplementary material for: Reduced CD5 on CD8+ T Cells in Tumors but Not Lymphoid Organs Is Associated With Increased Activation and Effector Function
Source: Front Immunol. 2021 Jan 28;11:584937. doi: 10.3389/fimmu.2020.584937 (PMC7876331; doi:10.3389/fimmu.2020.584937)
Supplement: Supplementary Figure 1 — Gating strategy for CD5high and CD5-/low T cell. Cells were gated based on their forward scatter and side scatter. Duplicate and dead cells were excluded then CD3+ cell were gated followed by CD4- and CD8- cell gating. CD5 on each CD4+ T cells and CD8+ T cells was gated 30% from the right as high expression and 30% from the left as low expression. Further analyses were carried out to look at CD69 and PD-1 surface markers. [file Presentation_1.pptx]

## Slide 1
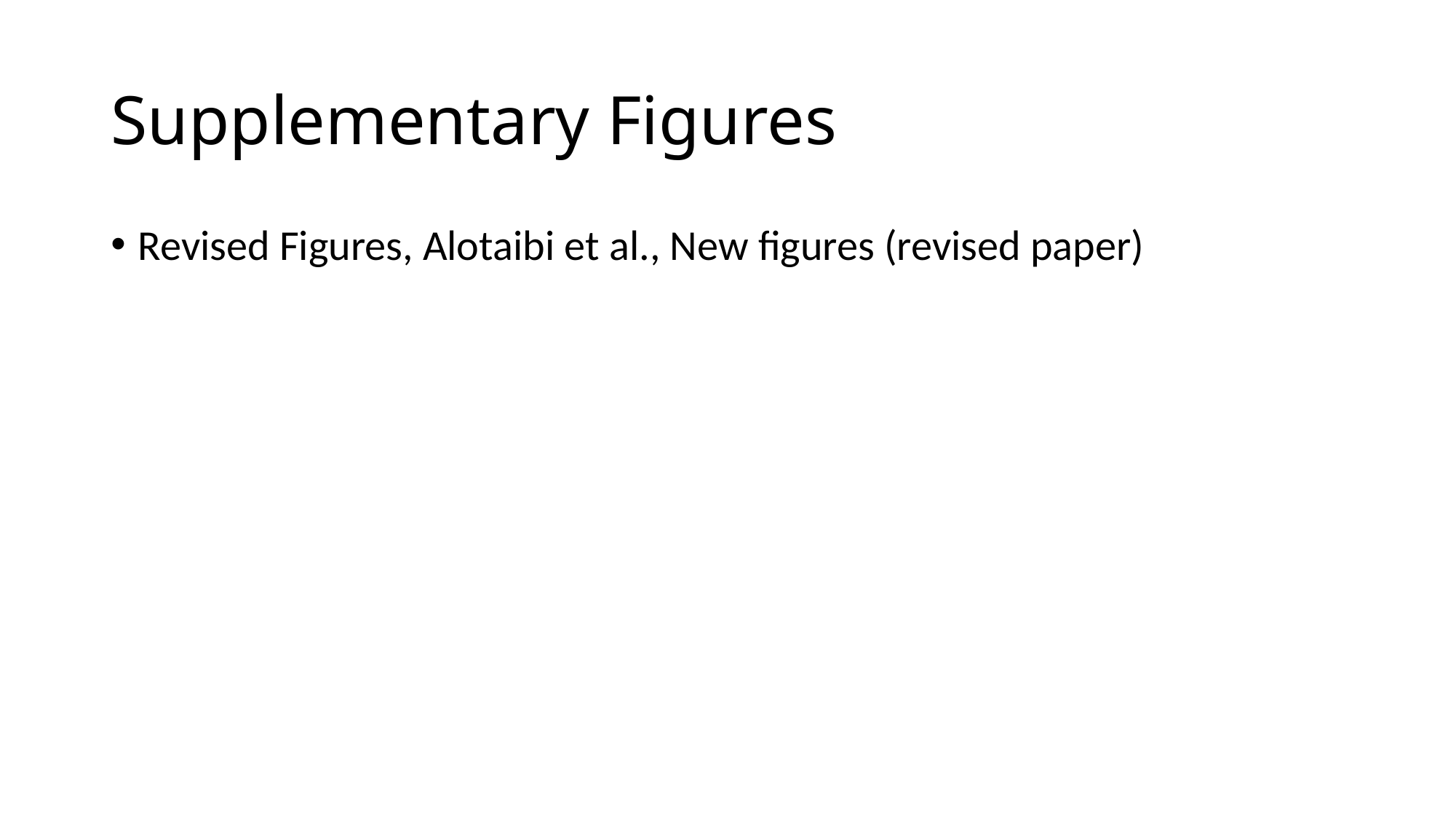

# Supplementary Figures
Revised Figures, Alotaibi et al., New figures (revised paper)

## Slide 2
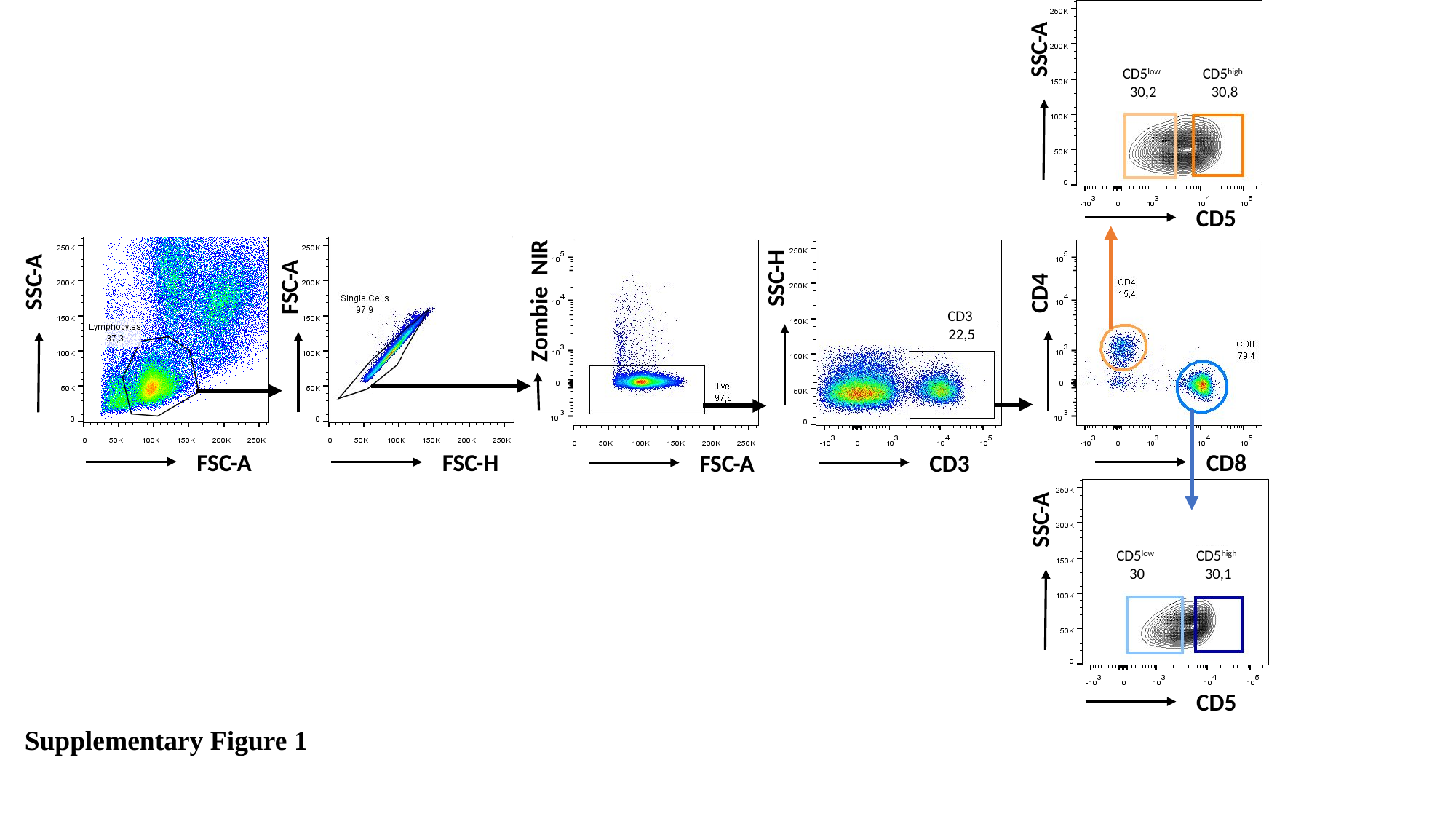

SSC-A
CD5low
30,2
CD5high
30,8
CD5
SSC-H
SSC-A
CD4
FSC-A
Zombie NIR
CD3
22,5
FSC-A
CD8
FSC-H
CD3
FSC-A
SSC-A
CD5low
30
CD5high
30,1
# Supplementary Figure 1
CD5

## Slide 3
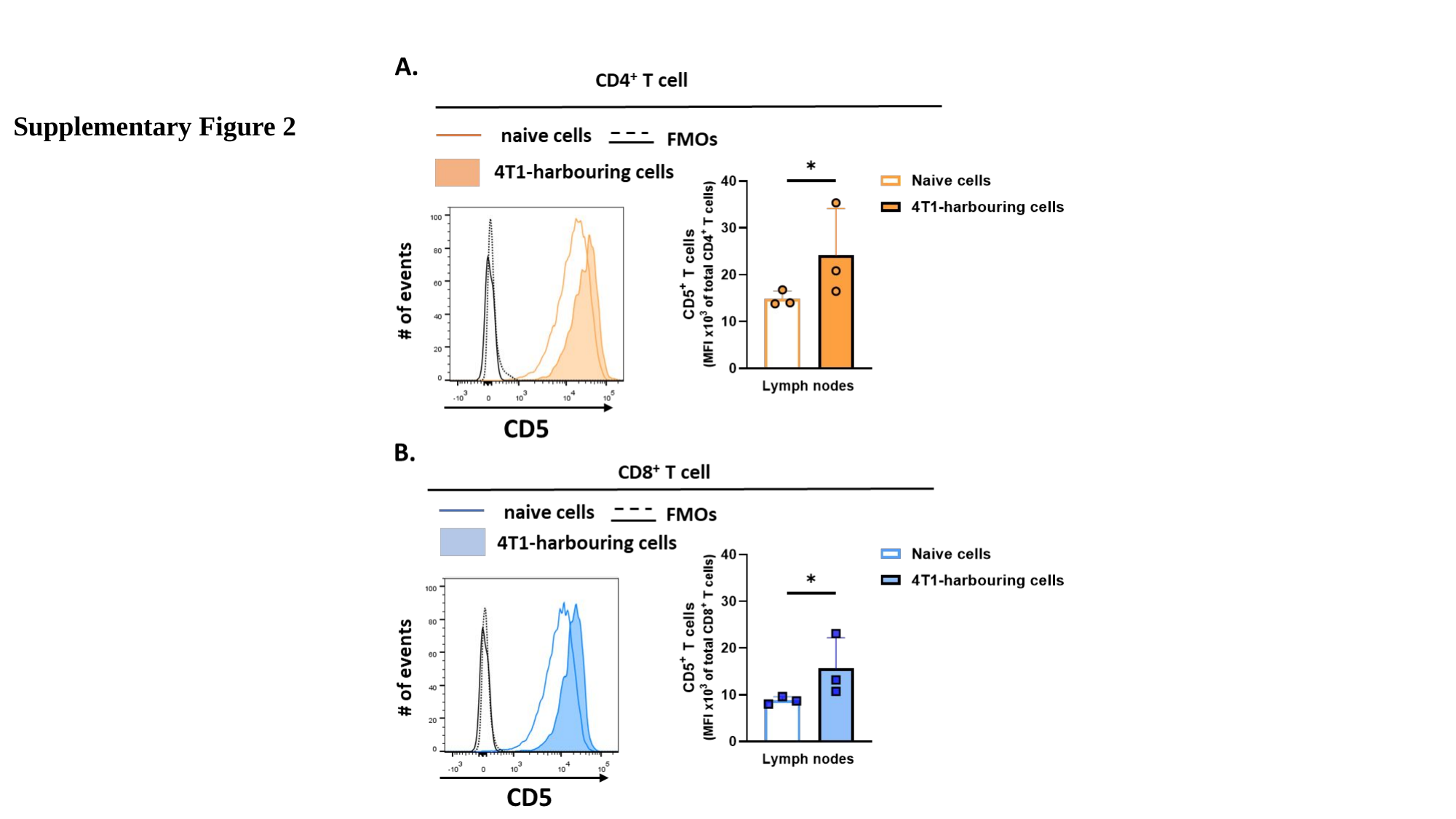

Supplementary Figure 2

## Slide 4
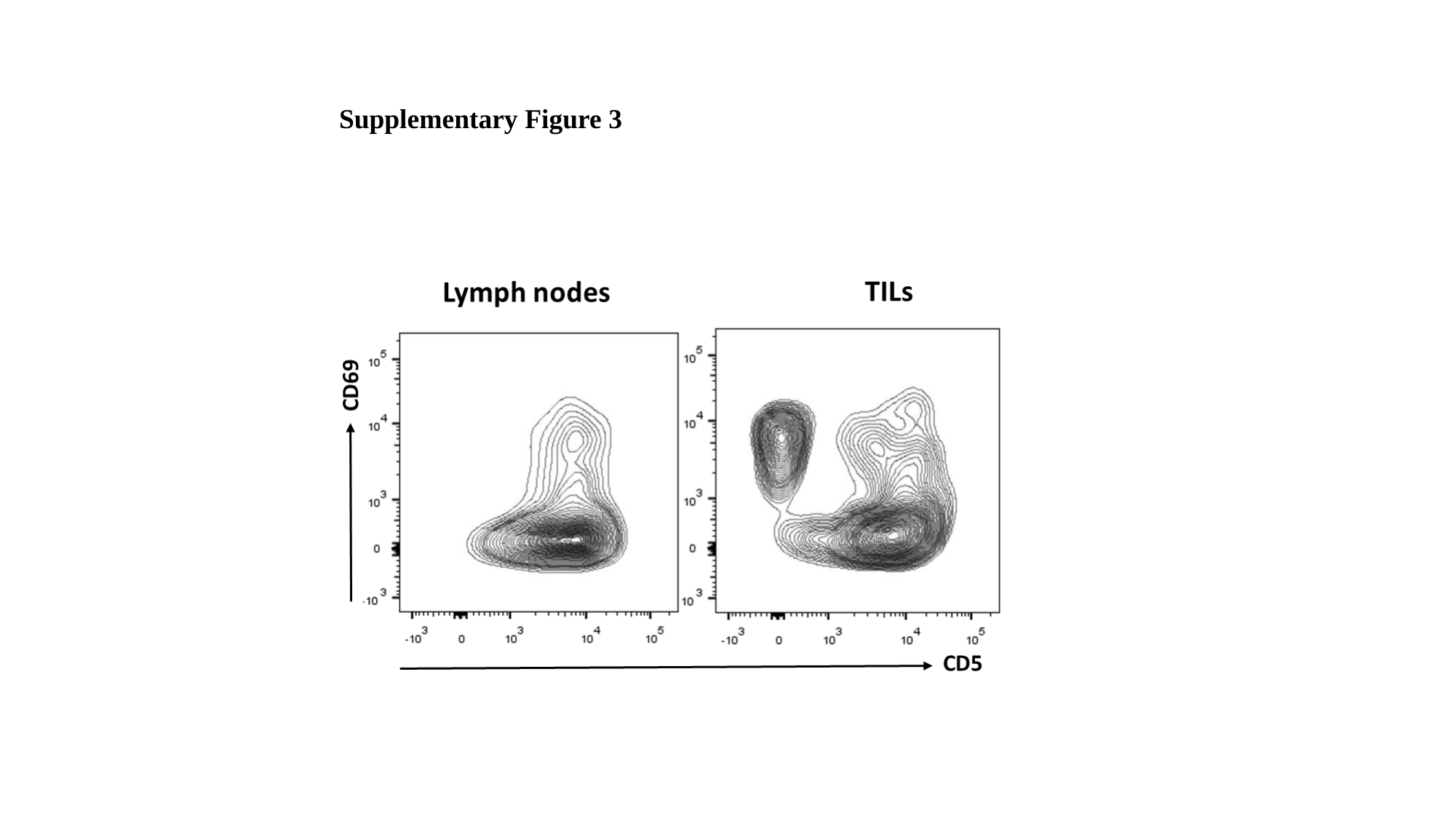

Supplementary Figure 3

## Slide 5
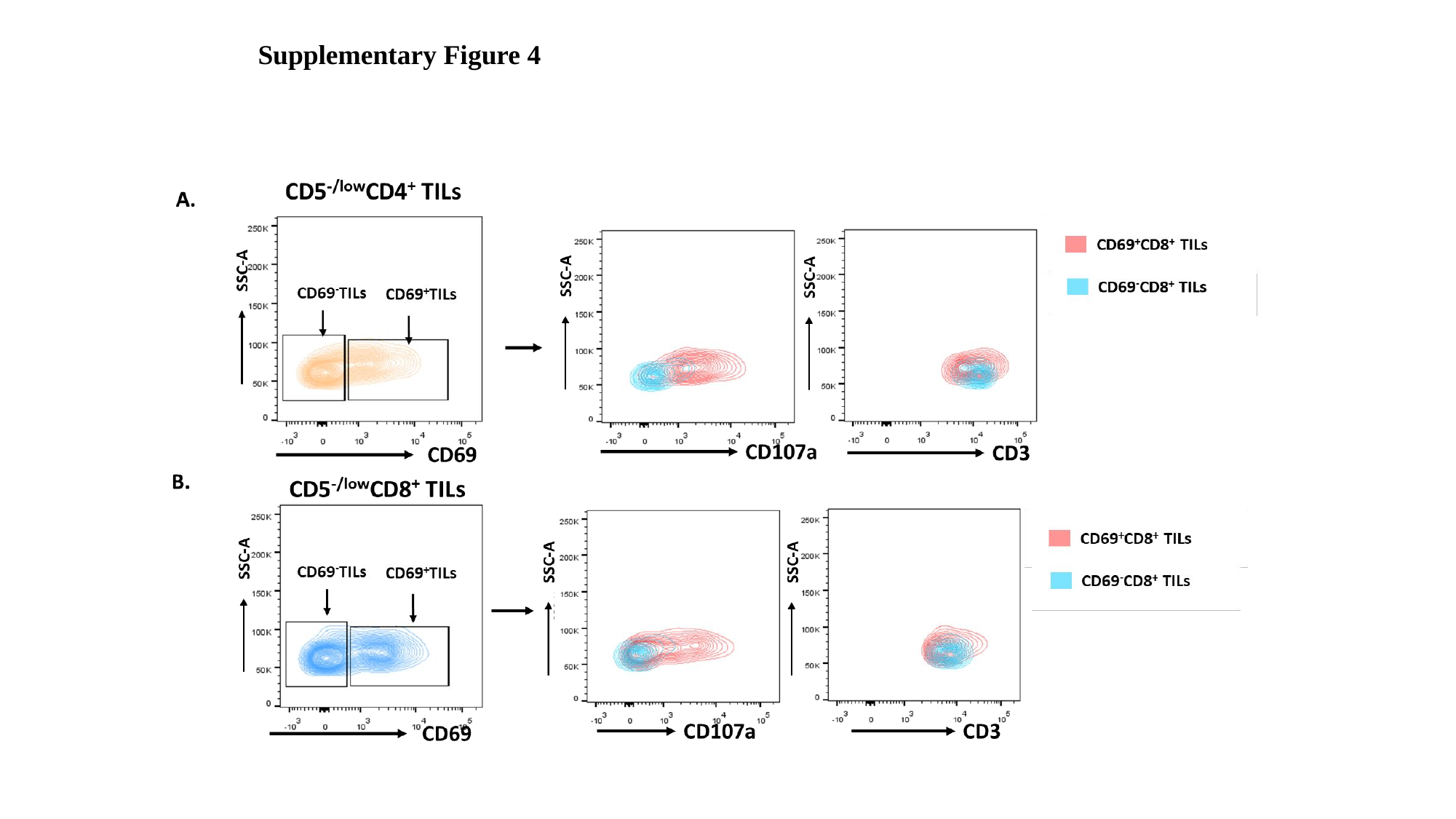

Supplementary Figure 4
